# One-Pot Preparation of Metal–Polymer Nanocomposites in Irradiated Aqueous Solutions of 1-Vinyl-1,2,4-triazole and Silver Ions

**DOI:** 10.3390/polym13234235

**Published:** 2021-12-03

**Authors:** Alexey A. Zezin, Alexey A. Zharikov, Artem I. Emel’yanov, Alexander S. Pozdnyakov, Galina F. Prozorova, Sergei S. Abramchuk, Elena A. Zezina

**Affiliations:** 1 Department of Chemistry, Lomonosov Moscow State University, 119991 Moscow, Russia; aazezin@yandex.ru (A.A.Z.); garikov-aleksey@mail.ru (A.A.Z.); abr@polly.phys.msu.ru (S.S.A.); zezi-len@yandex.ru (E.A.Z.); 2 Enikolopov Institute of Synthetic Polymeric Materials, Russian Academy of Sciences, Profsoyuznaya St., 70, 117393 Moscow, Russia; 3 Favorsky Irkutsk Institute of Chemistry, Siberian Branch of the Russian Academy of Sciences, Favorsky St., 1, 664033 Irkutsk, Russia; emelyanov@irioch.irk.ru (A.I.E.); prozorova@irioch.irk.ru (G.F.P.)

**Keywords:** radiation-induced reduction, one-pot synthesis, poly(1-vinyl-1,2,4-triazole), metal–polymer nanocomposites, silver nanoparticles

## Abstract

Metal–polymer nanocomposite polyvinyltriazole–silver nanoparticles were obtained using one-pot synthesis in irradiated aqueous solutions of 1-vinyl-1,2,4-triazole (VT) and silver ions. Gel permeation chromatography data show that upon radiation initiation, the molecular weight of poly(1-vinyl-1,2,4-triazole) increases with increasing monomer concentration. To study the kinetics of polymerization and the features of the radiation–chemical formation of nanoparticles, UV-Vis spectroscopy was used. TEM images show a relatively small average size of the forming nanoparticles (2–3 nm) and a narrow size distribution, which shows the effective stabilization of nanoparticles by triazole substituents at a molar ratio of VT and silver ions of 25/1. The addition of ethyl alcohol was used to increase the efficiency of synthesis and suppress the crosslinking of macromolecules in solution. The results of the work show that aqueous–alcoholic solutions of 1 wt.% VT can be used to obtain soluble nanocomposite materials. 10 wt.% monomer solutions have prospects for use in the preparation of polymer gels filled with nanoparticles.

## 1. Introduction

Metal–polymer nanocomposites may serve as the basis of functional materials possessing unique optical, magnetic, catalytic, and bactericidal properties, and materials for selective drug delivery. Therefore, the approaches to their synthesis keep intensively developing [1,2,3,4,5,6,7,8]. In recent years, great efforts have been invested in the development of one-stage methods for the production of nanocomposites, in which the synthesis of metal nanoparticles or metal oxides and the formation of a stabilizing matrix occurs in one reactor [1,2,3,4,5,6,9,10,11,12]. Radiation–chemical approaches are widely employed for the synthesis and modification of advanced polymer materials [6,13,14,15,16,17,18]. The possibility to change the radiation parameters such as dose rate and total absorbed dose over a wide range ensures unique opportunities for obtaining materials with tuned properties. Radiation-induced reduction results in the assembly of nanoparticles of a controlled size without impurities [6,15,16,18], which is especially important for medical and catalytic purposes. Radiation-initiated polymerization permits the obtaining of materials without residues of initiators or catalysts and makes it possible to carry out reactions without heating [13,17]. Thus, radiation–chemical approaches provide unique opportunities for the synthesis of both nanoparticles and polymer matrices in one reactor.

Silver nanoparticles can be used in biomedical imaging and photothermal therapy such as cancer therapy. AgNPs exhibit different cytotoxicity and have different effects on cellular mechanisms, depending on the chemical composition of the surface and concentration [19,20]. Nanocomposites also show thermo-responsive antibacterial properties [21,22]. The nanocomposites containing silver nanoparticles are prospective materials for optical and catalytic systems [1,15,16,18,23]. However, research attention was mainly paid to the production of biocidal materials, since the application of metal–polymer nanocomposites (unlike antibiotics) did not lead to bacterial resistance [24,25,26]. Poly(1-vinyl-1,2,4-triazole) (PVT) is a beneficial matrix for the synthesis of silver nanoparticles due to the high ability of macromolecules to bind silver ions and stabilize metal nanoparticles. Among the important properties of this polymer are pharmacological activity, nontoxicity, and resistance to aggressive media. Silver nanoparticles incorporated into the PVT matrix exhibit high antibacterial, immunomodulatory, and cytotoxic activity, enhance body defenses, and exert a protective effect against the Yersinia pestis bacteria and anthrax [27,28,29].

PVT-based nanocomposites with silver nanoparticles were previously synthesized by the chemical reduction of silver ions in an aqueous solution of PVT using different reducing agents (sodium borohydride, formaldehyde, etc.) [30], which complicated production and required laborious purification of the target nanocomposites from impurities. Thus, it is relevant to use eco-friendly and one-stage methods using radiation–chemical approaches for the synthesis of metal–polymer composites. Earlier, we successfully employed X-ray radiation for the preparation of film and soluble metal–polymer nanocomposites [12,18,31,32]. The possibility of obtaining hybrid materials in irradiated 0.1 wt.% aqueous solutions of 1-vinyl-1,2,4-triazole (VT) containing gold ions [12] was previously demonstrated using UV-visible spectroscopy. In the present work, we systematically examined the conditions for the formation of PVT-Ag nanocomposites in solutions with a monomer content of 1 and 10 wt.% under X-ray irradiation.

## 2. Materials and Methods

The starting monomer 1-vinyl-1,2,4-triazole (VT) was synthesized according to the published method [33] using 1,2,4-triazole. For the preparation of the samples, the following reagents were employed: silver nitrate, ethanol (all of the analytical grade, Sigma-Aldrich, Munich, Germany), and distilled water (solvent). PVT–Ag nanocomposites were synthesized in 1 and 10 wt.% VT solutions with a molar ratio of VT and Ag^+^ ions = 25:1. The suspensions were prepared by mixing VT aqueous solutions with silver nitrate.

The X-ray irradiation was carried out at 293 K using a 5-BKhV-6W tube with tungsten anode (applied voltage 45 kV, anode current 80 mA) in polymer test tubes of 5 mm diameter. Such conditions ensured the uniform generation of radiolysis products. To prevent the oxidation of the formed nanoparticles with air oxygen dissolved in the aqueous medium, before irradiation the sample was bubbled with pure argon (99.9999%). The dose rate either of 19 Gy/s or 6.2 Gy/s was measured using a ferrosulfate dosimeter irradiated in the same geometry as the samples studied. For metal–polymer complexes the actual dose rate was calculated taking into account the mass absorption coefficients for X-rays with an effective energy of 20 keV, which provides a reasonable representation of the actual X-ray spectrum. The irradiation was carried out in water and a water–alcohol mixture containing 10 vol.% of ethanol.

The structure of the prepared nanocomposites was studied on a Leo-912AB OMEGA transmission microscope (Carl Zeiss, Oberkochen, Germany) with a resolution of 0.3 nm. The optical spectra were measured on UV- and Perkin Elmer Lambda 9 instruments (the optical range was 200–900 nm, Überlingen, Germany).

A Shimadzu LC-20 Prominence system (Shimadzu Corporation, Kyoto, Japan), equipped with a differential refractive index detector Shimadzu RID-20A, was used to determine the molecular weight of the polymer by gel permeation chromatography (GPC). Chromatographic analysis was performed at 50 °C, with used N, N-dimethylformamide (DMF) as the eluent at a flow rate of 1 mL/min. An Agilent PolyPore 7.5 × 300 mm (PL1113-6500) column was used. The samples were dissolved for 24 h with stirring at 50 °C. Calibration was performed using a set of polystyrene standards, consisting of 12 samples with molecular weights ranging from 162 to 6,570,000 g/mol (Polystyrene High EasiVials PL2010-0201).

## 3. Results

**Figure 1:**
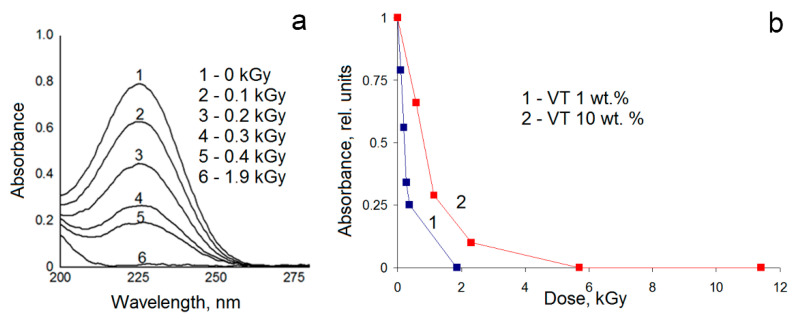
The UV-VIS spectra of irradiated 1 wt.% 1-vinyl-1,2,4-triazole (VT) solutions and diluted by a factor of 150 (**a**); the dependence of the intensity of the adsorption band at 226 nm (rel. units) on the absorbed dose (kGy) for 1 and 10 wt.% VT solutions (**b**).

First, we studied the peculiarities of the radiation-initiated polymerization in aqueous solutions of VT. The absorption band with a maximum at 226 nm was assigned to the double bond of the monomer molecules [12]. The data of UV-vis optical spectroscopy show that the irradiation of 1 wt.% solutions of VT leads to a gradual decrease in the absorption band with a maximum at 226 nm (Figure 1a). The analysis of optical spectroscopy data does not detect the presence of a monomer for absorbed doses of more than 2 kGy, which indicates the completion of monomer polymerization (Figure 1b). A similar effect is observed for irradiated 10 wt.% solutions of VT, (Figure 1b); in this case, a decrease in the concentration of the monomer occurs at higher doses and its band becomes invisible for the absorbed dose of 5.5 kGy.

GPC data exhibit that for 1 wt.% solutions of VT (Table 1), irradiation leads to the formation of macromolecules with a weight average molecular weight (Mw) from 8.2 × 10^4^ to 1 × 10^5^ Da, depending on the radiation dose.

It should be noted that GPC gives an essential error for determining the molecular weight of crosslinked polymers. However, this method allowed us to carry out comparative studies of the effect of the radiation dose on the crosslinking processes.

**Figure 2:**
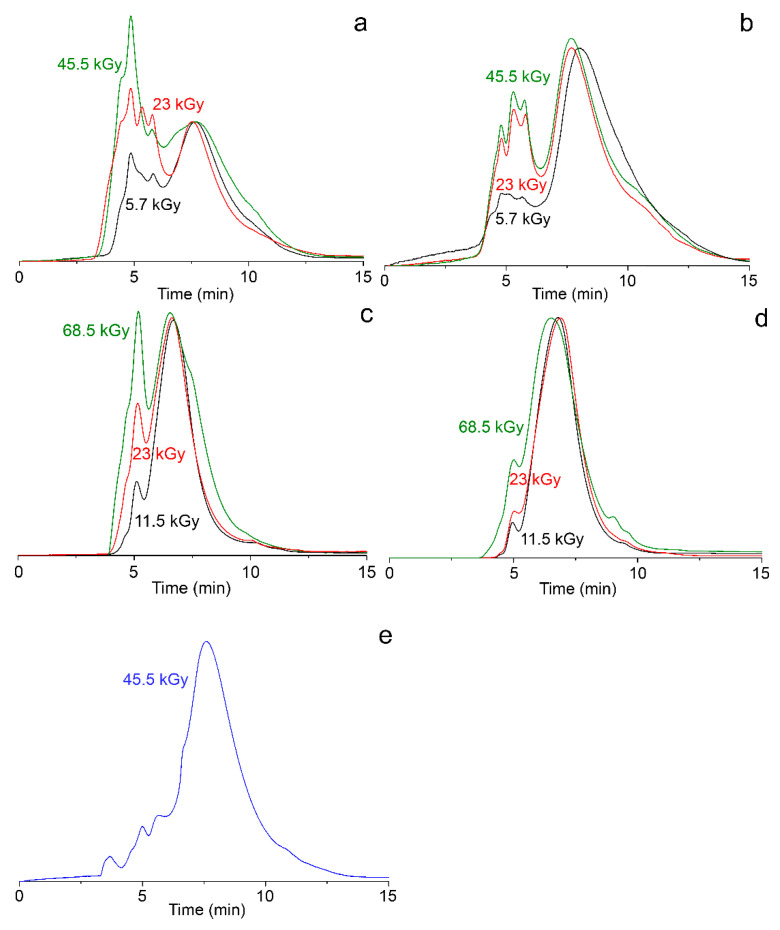
Chromatograms of irradiated samples: (**a**) 1 wt.% 1-vinyl-1,2,4-triazole (VT) solution; (**b**) 1 wt.% VT solution with 10 vol.% of ethanol; (**c**) 10 wt.% VT solution; (**d**) 1 wt.% VT solution with 10 vol.% of ethanol; (**e**) 1 wt.% VT solution containing Ag^+^ ions with 10 vol.% of ethanol.

The occurrence of products with a high molecular weight (more than 8 × 10^5^ Da) is also observed. The fraction of high-molecular-weight products increases with the growth of absorbed dose (Figure 2a). In irradiated 10 wt.% solutions of VT (Figure 2c), products with Mw from 5.5 × 10^5^ to 7.6 × 10^5^ Da (Table 1) and products which exceed 2 × 10^6^ Da are formed.

The irradiation of VT in aqueous-alcoholic solutions significantly decreases the content of high-molecular-weight fractions in the synthesized polymer. The fraction of PVT with Mw from 6.5 × 10^5^ to 7.9 × 10^5^ Da and from 5.5 × 10^4^ to 7.8 × 10^4^ Da is the predominant product for solutions containing 10 and 1% of VT, respectively.

˙OH radicals belong to the main products of water radiolysis [6,16]. On the one hand, they effectively initiate polymerization processes [13,17]; on the other hand, ˙OH radicals are oxidizing agents of metal ions and clusters [15,16] (intermediates of nanoparticle formation). For this reason, polymerization processes have been investigated also in the presence of ethanol additives, which are an effective acceptor of ˙OH radicals. The irradiation of monomers in water–alcohol solutions leads to a significant decrease in the fraction of high-molecular-weight compounds. The polymer fraction with a lower molecular weight becomes the predominant product for solutions containing both 1 and 10 wt.% VT solutions (Figure 2b,d).

One-pot synthesis of metal–polymer nanocomposites PVT/Ag was carried out in 1 and 10 wt.% VT solutions containing 10 vol.% ethanol at a molar ratio of Ag/VT = 1/25. In irradiated aqueous–alcoholic solutions with 1 wt.% of VT, containing silver ions (Figure 2e), along with the main product, the higher-molecular-weight compounds were also formed. The irradiation of 10% VT solutions with silver ions gave insoluble products of liquid (amorphous) hydrogels.

**Figure 3:**
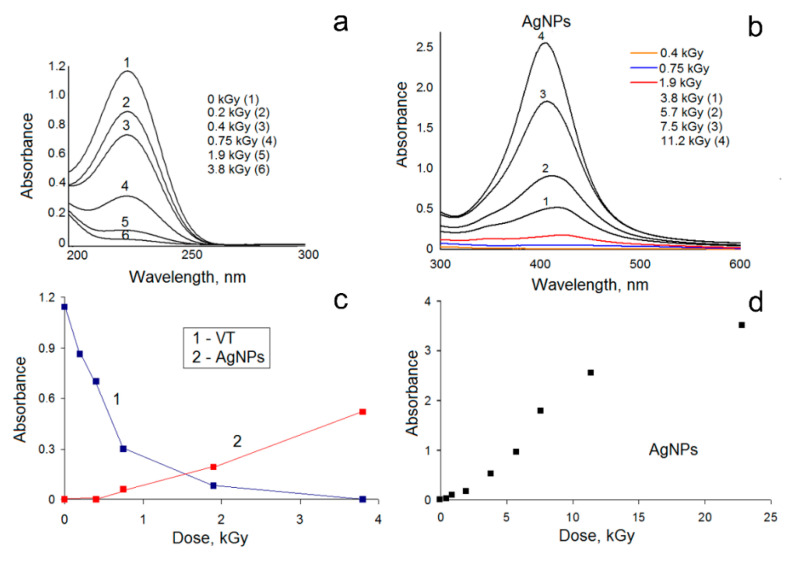
The UV-VIS spectra of irradiated 1 wt.% 1-vinyl-1,2,4-triazole (VT) solutions containing Ag^+^ ions with 10 vol.% ethanol in the range from 200 nm to 300 nm (diluted by a factor of 100) (**a**) and in the range from 300 to 600 nm (**b**); the dependence of the intensity of the adsorption bands at 226 nm and silver nanoparticles (AgNPs) plasmon bands on the absorbed dose up to 4 kGy (**c**); the dependence of the intensity of the silver nanoparticles plasmon bands on the absorbed dose (**d**).

For 1 wt.% VT solutions containing silver ions, the polymerization processes finished after irradiation up to dose 4 kGy (Figure 3a,c). Irradiation leads to a gradual increase in the intensity of the absorption bands of silver nanoparticles (Figure 3b,d). The maximum intensity of the absorption band reaches a maximum at 405 nm in a solution irradiated to a dose of 24 kGy. In the case of a 10 wt.% solution of a monomer with silver ions, polymerization processes are mainly completed in the range of absorbed doses up to 7 kGy. An increase in the radiation dose also results in a gradual increase in the intensity of the optical signal of nanoparticles. However, irradiation to a dose of 68 kGy and above leads to the formation of an insoluble product, which makes it impossible to perform quantitative spectroscopic measurements and receive information about the formation of nanoparticles with significant conversions of silver ion reduction.

**Figure 4:**
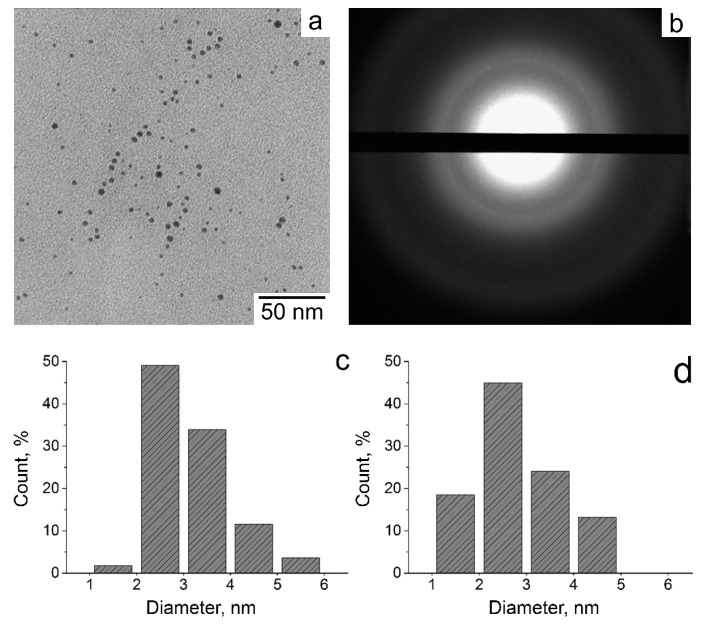
The TEM image (**a**), the microdiffractogram (**b**), and the size distribution (**c**) of silver nanoparticles (AgNPs) obtained in the irradiated up to a dose of 24 kGy 1 wt.% 1-vinyl-1,2,4-triazole (VT) solution, molar ratio of Ag^+^ ions/VT = 1/25; the size distribution (**d**) of AgNPs obtained in the irradiated up to a dose of 24 kGy 10 wt.% (VT) solution, molar ratio of Ag^+^ ions/VT = 1/25.

Analysis of the TEM images (Figure 4a) shows the presence of nanoparticles from 1 to 6 nm in size in the irradiated samples of 1 wt.% VT solutions containing silver ions. The selected area electron diffraction pattern (SAEDP) (Figure 4b) proves that the nanoparticles obtained are Ag with a face-centered cubic crystal structure. The reflections observed correspond to the interplanar distances of 2.35, 2.04, 1.45, and 1.23 Å (Figure 4b), which perfectly match the Ag face-centered cubic crystal lattice [34]. The irradiation of 10 wt.% VT also leads to the obtaining of relatively small nanoparticles with a narrow size distribution.

## 4. Discussion

The products of water radiolysis play the major role in the above polymerization and formation of nanoparticles, since water is the main component of the system (80 vol.% or more for the studied systems). For solutions with relatively high concentrations of monomers and ethanol, one should also take into account the direct action of irradiation on VT and ethanol, which affords additional radical products. The process of water radiolysis can be represented as follows: [16]

H_2_O → e_aq_^−^, ˙OH, H_3_O^+^, H˙, H_2_, H_2_O_2_, HO_2_˙ (1)

**Scheme 1:**
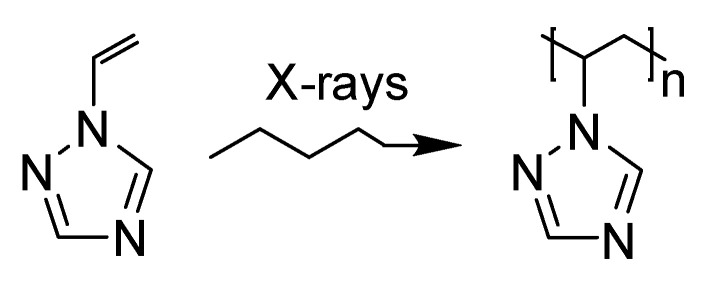
Polymerization of 1-vinyl-1,2,4-triazole.

The action of ionizing irradiation gave ˙OH radicals and hydrated electrons in the highest yields [15,16]. Extremely active ˙OH radicals react instantly with any oxidizable molecule in the immediate surroundings and can initiate radical polymerization (Reaction (2), Scheme 1). Atomic hydrogen can react in the same way (Reaction (3)): further propagation (Reaction (4)) and termination (Reaction (5)) of the macromolecular chain lead to the end of polymerization and obtain a polymer (Table 1).

˙OH + M → HOM˙(2)

H˙ + M → HM˙ (3)

HOM˙ (HM˙) + M → HOMM˙ (HMM˙) + M →…. → HO(M)_n-1_M˙ (H(M)_n-1_M˙) (4)

R˙ + R˙ → R-R (R = H(M)_n_, HO(M)_n_) (5)

˙OH (˙H) + M_n_ → M˙_n_ + H_2_O (H_2_) (6)

M˙_n_ + M˙_m_ → M_n_ − M_m_ (7)

**Scheme 2:**
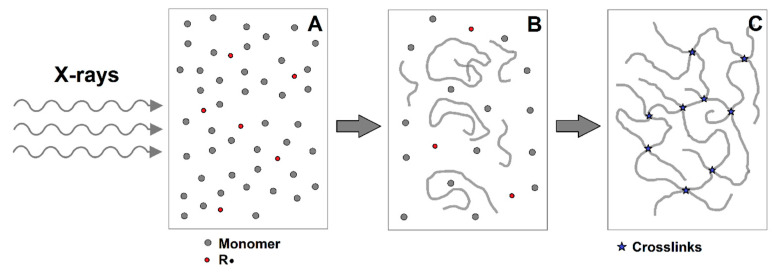
Radiation-initiated polymerization and crosslinking of macromolecules in aqueous solutions. (**A**)—chain propagation, (**B**)—chain termination, (**C**)—molecular crosslinking.

The reactions of ˙OH and ˙H radicals with macromolecules result in the abstraction of hydrogen atoms to furnish macroradicals (Reaction 6). Further recombination of macroradicals leads to an increase in the molecular weight of the polymer [6,13,14] (Reaction (7), Scheme 2B) and the formation of compounds with a higher molecular weight in the polymerization system. At high radiation doses, insoluble crosslinked products are ultimately formed (Scheme 2C).

Hydrated electrons possess extremely high reduction potentials (−2.9 V [35]). However, ˙OH radicals, acting as oxidants, react with small, charged Ag clusters that dramatically decrease the efficiency of nanoparticle formation [15,16]. To scavenge ˙OH radicals and ensure favorable conditions for the reduction of metal ions, alcohol additives are usually used. The generated alcohol radicals have reducing properties [15,16].

CH_3_CH_2_OH + ˙OH → CH_3_˙CHOH + H_2_O (8)

The additives of ethanol were used for experiments (Reaction (8); (E^0^_CH3˙CHOH_ −1.4 B [18,35]). The oxidation of alcohol radicals results in the formation of acetaldehyde, which acts as a weak reducing agent [18,31,36]. Thus, when ethanol is used as a scavenger of ˙OH radicals, only reducing products are generated in water–organic mixtures. Unlike ˙OH radicals, alcohol radicals are not able to participate in oxidative reactions of hydrogen atoms abstraction from organic compounds.

The features of the radiation–chemical formation of PVT were studied in both the presence and absence of ethanol additives. The radiation–chemical yield per 100 eV of absorbed energy (G) of the VT polymerization can be calculated using the initial (close to linear) sections of the dependencies between the intensity of the absorption band at 226 nm and the absorbed dose:

G = (9.65 × 10^6^ × ∆C)/(Dρ) (9)

where ∆C is the change in the monomer concentration (mol/L), D is the absorbed dose in Gy, and ρ is the density of the irradiated substance (close to 1). Data on the yields of monomer conversion under the irradiation are given in Table 2. Comparison of the yields of monomer transformation in solutions shows that in 10 wt.% VT solutions, conversion occurs 2.5–3 times faster than in 1 wt.% solutions. A logical explanation for this effect is the competition between initiation, the subsequent chain propagation reactions (Reactions (2) and (3)), and the recombination of radicals (Reaction (5)). An increase in the monomer content enhances the probability of chain initiation and propagation processes.

In this case, the overwhelming majority of initiation processes should be triggered by the reactions involving alcohol radicals (Reaction (10)).

We also found that the application of 10 vol.% ethyl alcohol additives in the polymerization of VT in irradiated aqueous solutions does not have a noticeable effect on the kinetics of the radiation-initiated polymerization and the yields of the monomer (Table 2). Therefore, the transformation of ˙OH radicals into alcohol radicals (Reaction (8)) has no significant influence on the efficiency of the initiation processes. The 10 wt.% VT solutions contain comparable concentrations of monomer and alcohol and, according to a rough estimate, the reactions of ˙OH radicals with the monomer and ethanol should have a comparable probability. The effect of initiation by alcohol radicals is most pronounced for 1 wt.% monomer solutions in which the alcohol concentration exceeds the VT concentration by 10 times. In this case, the overwhelming majority of initiation processes should be triggered by the reactions involving alcohol radicals (Reaction (10)).

CH_3_˙CHOH + M → CH_3_CHOH M˙ + M →…. →(M)_n_M˙ (10)

In solutions containing ethyl alcohol and silver ions, some decreases in the yields of radiation-initiated polymerization are observed (Table 2), which may be explained by the competition of the reactions of alcohol radicals in the processes of the initiation of polymerization and the reduction of silver ions.

Analysis of the experimental data shows that the initial concentration of the monomer affects the molecular weight of the target PVT. In the solution irradiated up to the dose of 11.5 kGy 10 wt.% VT, the Mw of the main product is 6.6 × 10^5^ Da (Table 1). As seen from Table 1, after the completion of VT polymerization in an irradiated 1 wt.% solution, polymers with significantly lower molecular weight are formed, which correlates with a decrease in radiation-induced polymerization yields. However, the significant influence on the molecular weight seems to be the competition of the processes of the initiation of polymerization/chain propagation and the recombination of radical particles (Reaction (5)). Initiation and propagation rates can be represented by the equations:

V_in_ = k_in_[R˙][M] (11)

V_p_ = k_p_[R(M)_n_˙][M] (12)

The recombination rate is proportional to the squared stationary concentrations of radical particles:

V_r_ = k_r_[R˙]^2^ (13)

The generation of radical species in solutions under irradiation occurs at a constant rate [13,17] or changes slightly with some changes in the chemical composition and structure of the irradiated system. Analysis of the Equations (11) and (12) demonstrates that the efficiency of polymerization processes increases in comparison with recombination in solutions with a growth monomer content, which should lead to the production of macromolecules with a higher macromolecular mass.

When 10 vol.% additives of ethanol additives are used in the polymerization of VT in irradiated aqueous solutions, the molecular weight of the target products becomes somewhat lower, while the content of products with a relatively high molecular weight significantly decreases both in samples containing 1 and 10 wt.% VT (Figure 2b,d). The reduction in molecular weights of the formed polymers can be explained by chain transfer reactions. The most likely participants of this process (Reaction (14)) may be the additives of ethanol.

R(M)_n_M ˙ + CH_3_CH_2_OH + → → R(M)_n_MH + CH_3_˙CHOH (14)

As seen from chromatograms, along with the formation of the major product, compounds with molecular weights of more than 2 × 10^6^ and 8 × 10^5^ Da are detected for irradiated solutions of 10 and 1 wt.% VT, respectively. The relative content of these products increases with the growth of the absorbed radiation dose in 10 and 1 wt.% VT solutions. The molecular weight is augmented due to the recombination of macroradicals (Reaction (7), Scheme 2), formed via the reactions of ˙OH radicals with macromolecules (Reaction (6)). The content of these high-molecular-weight compounds sharply decreases in solutions containing ethanol, owing to the fact that the additives of ethyl alcohol lead to an efficient scavenging of ˙OH radicals [16].

Silver nanoparticles were obtained in irradiated aqueous solutions containing ethanol additives for the scavenging of ˙OH radicals, which act as oxidizers for silver clusters. Attempts to carry out synthesis in a 1 wt.% solution of VT without ethanol additives led to a multiple decrease in the efficiency of the generation of silver nanoparticles. The formation of insoluble products due to the crosslinking of macromolecules (Reactions (6) and (7)) hindered the study of nanocomposites in aqueous solutions of 10 wt.% VT. The mechanisms of the radiation–chemical formation of silver nanoparticles were studied in detail in works [37,38,39,40]. The process starts with the reduction of silver ions to isolated atoms and the formation of the clusters:

Ag^+^ + e^−^_aq_ → Ag^0^ (15)

Ag^0^ + Ag^+^ → (Ag_2_)^+^ →…. → (Ag_m_)^n+^ (16)

The growth of clusters upon the reduction of silver ions on their surface (Reaction (17)) and coalescence processes (Reaction (18)) lead to the formation of nanoparticles and their enlargement.

(Ag_m_)^n+^ + (Ag_k_)^g+^ → (Ag_m__+__k_)^(^^n+^^g)+^ (17)

(Ag_m_)^n+^ + Ag^+^ → (Ag_m+1_)^n+1^ + e^-^_aq_ (CH_3_˙CHOH, acetaldehyde) → (Ag_m+1_)^n^ (18)

Nucleation reactions are limited to the processes involving the strongest reducing agent. E^0^(Ag^+^/Ag^0^) = −1.8 V [35], therefore, the reduction of silver ions to isolated atoms and the formation of small clusters (Reactions (15) and (16)) can be ensured by a hydrated electron [38,39,40]. The reduction of silver ions on the surface of nanoparticles, leading to the growth of nanoparticles (Reaction (17)), can occur due to reducing agents having lower reduction potentials, including acetaldehyde [18,31,36].

**Scheme 3:**
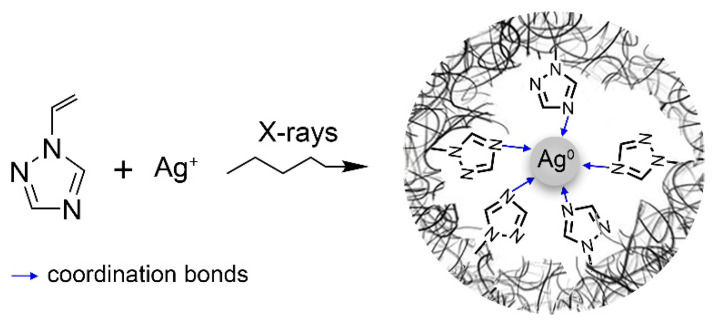
Synthesis of the nanocomposite with AgNPs in PVT.

The stabilization of metallic silver nanoparticle is provided by the coordination bonds of triazole rings (predominantly with nitrogen atoms in the fourth position, since it is the most electronegative) with silver surface atoms [41]. In this case, the resulting bond between the polymer and the nanoparticles will be greatly enhanced by cooperative multipoint coordination bonding simultaneously with many surface atoms (Scheme 3).

The formation of nanoparticles via the reduction of silver ions can be described in terms of a zero-order reaction, since it takes place upon the generation of radiolysis products at a constant rate. However, analysis of the intensities of nanoparticle plasmon signals shows that the formation of nanoparticles is complex: at the initial stages, the rate of their formation is much lower. Previously, a similar dependence was observed for the generation of silver nanoparticles in irradiated water–alcohol dispersions of PVT containing silver ions [42]. At the initial stage (up to 3–4 kGy for 1 wt.% VT solution), the nucleation processes predominate over the assembly of nanoparticles. In this case the oxidation of labile clusters plays an important role, which results in a decrease in the effective rate of silver ions reduction and, consequently, in a decrease in the efficiency of nanoparticles generation. As nanoparticles are being formed, the adsorption and reduction of ions on the Ag surface begin to be major processes. This leads to an increase in the effective rate of nanoparticle formation due to the fact that not only a hydrated electron, but also weaker reducing agents (alcohol radicals and acetaldehyde), are involved in the reduction.

The molecular weight of PVT obtained in irradiated aqueous–alcoholic solutions containing silver ions is 2 × 10^5^ Da, which is significantly higher than for PVT (Table 1) synthesized under similar conditions without silver ions. This result may be explained by the participation of alcohol radicals in the processes of the reduction of clusters and silver nanoparticles (Reaction (18)), which reduces the rate of the initiation of polymerization (Reaction (10)) and the likelihood of the participation of alcohol radicals in the chain transfer processes (Reaction (14)). However, the detected effect can be attributed not only to the influence of silver ions/nanoparticles on the polymerization conditions, but also to the intermolecular crosslinking of individual PVT macromolecules by nanoparticles into aggregates. Previously, the formation of associates was detected in poly(N-vinylimidazole) composites with copper nanoparticles [43].

## 5. Conclusions

In conclusion, it was found that metal–polymer nanocomposites PVT/AgNPs can be synthesized via a one-pot manner in irradiated solutions containing the monomer and silver ions. The application of aqueous–alcoholic solutions is crucial for the synthesis, both in terms of the efficiency of the nanoparticle formation and the control of PVT molecular weight. 

It is established that when aqueous–alcoholic solutions of VT with a molar ratio of monomer and silver ions of 25/1 are used for the synthesis, during polymerization, nucleation processes prevail. The radiation-induced generation of nanoparticles occurs mainly after the formation of macromolecules. 

The results obtained show that irradiation leads to the formation of relatively small nanoparticles (average size 2–3 nm) with a narrow size distribution both for 1 and 10 wt.% VT solutions. Thus, the application of VT, which under irradiation transforms to PVT, provides evidence that the triazole groups effectively interact with the clusters and surface of silver nanoparticles. 

The application of 1 wt.% monomer solutions allows soluble nanocomposites to be obtained. In the case of 10 wt.% solution, even in the presence of an alcohol, the crosslinking of macromolecules delivers insoluble products, which opens prospects for the development of one-pot methods for the synthesis of gels with metal nanoparticles.

## Figures and Tables

**Table 1:** Effect of 1-vinyl-1,2,4-triazole (VT) concentration and radiation dose on poly(1-vinyl-1,2,4-triazole) molecular weights.

VT Content, wt.%	Ethanol Content, vol.%	Irradiation Dose, kGy	Mw, kDa	Content, %
1	0	5.7	94	73
		23	101	54
		45.5	82	48
	10	5.7	55	84
		23	75	62
		45.5	78	61
10	0	11.5	760	91
		23	654	79
		68.5	550	69
	10	11.5	660	97
		23	655	96
		68.5	790	89

**Table 2:** Radiation–chemical yields of 1-vinyl-1,2,4-triazole (VT) polymerization in solutions.

Sample Composition	1 wt.% of VT	1 wt.% of VT, 10 vol.% of Ethanol	1 wt.% of VT, 10 vol.% of Ethanol, Ag^+^ Ions	10 wt.% of VT	10 wt.% of VT, 10 vol.% of Ethanol	10 wt.% of VT, 10 vol.% of Ethanol, Ag^+^ Ions
G of VT conversion/100 eV	2.3 × 10^3^	2.4 × 10^3^	1.6 × 10^3^	6.4 × 10^3^	6.3 × 10^3^	5.7 × 10^3^

## Data Availability

Not applicable.
